# Biomarker Development Study Publication Standards are Dead—Long Live Biomarker Development Study Publication Standards!

**Published:** 2007-02-03

**Authors:** James Lyons-Weiler

**Affiliations:** Cancer Informatics, Allison Park, Pennsylvania

At the University of Pittsburgh, I teach a graduate-level course ‘The Practical Analysis of High-Throughput Genomic and Proteomic Data’. 50% of the course grade is based on a paper project based on the re-analysis of published data sets. The aim of the project is to encourage the comparative evaluation of different approaches to the various analytic tasks for – omic based biomarker studies. The students are empowered by this course to understand – and to see for themselves – that different approaches to normalization, feature selection, and disease prediction model (a) exist, and (b) differ in their apparent relative performance in helping to generate lists of therapeutic targets or disease prediction models. We also learn about various data standards, mostly from the perspective of data formats, which are critical to re-analysis based algorithm evaluation studies.

Each time I teach the course, two or more students approach me with the following problem. They have found an interesting, peer-reviewed, published study, but the data from those studies are not available anywhere. I encourage them to write to the authors. When the authors fail to reply (which has been the mode response), I sometimes draft a follow-up letter or email requesting more information for access to the data to facilitate the students’ attempts to evaluate the many alternative approaches to analysis.

I am sad to say that both the reply rate to my own queries, and the success of acquiring data from such studies is disturbingly low.

There have been calls in the past to standardize the reporting of data from microarray studies (e.g. MAGE-ML and MIAME). These calls have been largely unsuccessful in terms of being adopted as publication standards (their original intent), and the proposed reporting standards have evolved into one of numerous possible ‘data standards’, with major investment being made within institutions, and by the National Cancer Institute to create ‘data standards’ for interoperable software for cancer research (e.g., caBIG™; von Eschenbach and Beutow, 2006).

While I do not at all eschew the use of model-based software development, or the creation of data standards for publication, it is my distinct impression and opinion that the original focus, publication and reporting standards, for microarray studies, and now for SNP, aCGH, proteomic profiling, and multiplexed biomarker studies, and clinical data, has been completely lost and forgotten. In my opinion, too much of the focus of biomedical informatics, and by far too much of the funding, has shifted from effort to understand how to best interpret the data from such studies, i.e., what should we do with the data, to how can we best manage the data, with the promise that ‘one day’ we’ll be better positioned to interpret the data. Expensive data management systems can place innumerable filters and sieves on the data prior to interpretive analysis and can sometimes restrict the range of analytic options, canalizing researchers to a narrow slice of possible discoveries, the limits of which are determined by software, not be expertise. Such practices could alter dramatically the apparent outcome of research studies that the system supports in ways that are at first not obvious. Institutions who purge the research focus of bioinformatics in favor of data management, who overbuild data management capabilities, who confuse Information Technology (IT) with Informatics, and who do not facilitate open discussions of alternative approaches to interpretation and analysis - do so at great peril to their long-term bottom line and risk failure in the short- and medium-term of their biomarker studies.

Recently, the incidence of research misconduct has trended upwards. This has been widely attributed to pressures placed on researchers to publish and win funding, and, I imagine, to the draw of personal financial stability that would accompany a blockbuster drug or assay. I think the trend it also due to the perception by researchers that their work is, somehow indemnified from critical evaluation by lax publication data reporting standards.

For these reasons, I enumerate here briefly calls for thought and action out of these concerns not from the data management/engineering IT perspective, but from a Clinical Translational Research Informatics perspective:

There is a very serious, evidently widespread, mistaken impression that the differences in the performance among various classifier-derived prediction models are trivial. Too few researchers report the performance measure of alternative prediction models, even when they have studied alternatives – a strategy and process that will impede widespread consensus of best practices of prediction modeling, and that, in individual studies, can ultimately prove self-defeating when the model that best predicts on one data fails to predict well with follow-up validation studies. It is far better in the early stages of biomarker-based prediction modeling studies to report on the performance of a range of models than to only report the most impressive, compelling or provocative result obtainable with a single study. Journals that publish biomarker development studies should require an appendix of all analytic results generate to date using the data upon which the highlighted results are based, and an overt statement from the authors attesting that all results have been reported.

Researchers are not sharing their data and no longer honor requests for data from their studies. For early detection and survivorship studies, research journals and governmental regulatory bodies should require the publication of all de-identified data that were used to derive conclusions in the course of a published study. Because this will vary from study to study, the trend toward full reporting of all data, and data standards-driven archival seems out of step with reality. Data from clinical covariates or factors not found to be significant fall into the category of ‘data used to derive conclusions in the course of a published study’, because the study concluded that those covariates and factor were not significant, and should be shared. Privacy issues do not exist after the data have been de-identified, and so requests not honoured due to patient privacy concerns should not be given any credence.

Reported data from biomarker studies should include (a) the raw data straight from its technological source, (b) any specific clinical data used to derive a conclusion from a study; (c) the survivorship studies precise survivorship data points for each patient. Patient privacy regulations in place can be met with these data sharing requirements. Publishing researchers should be prepared to stand behind and defend their published knowledge claims by providing data. The consequences of failing to hold ourselves to the highest standards are dire; I recently read reviewer comments of a grant proposal (my own, so I can share) that essentially claimed that past 25 years of published studies on individual protein and RT-PCR biomarker studies are too full of noise to warrant preselecting biomarkers from the literature. We must not be so intently focused on competing for priority scores that the products of our life sciences and biomedical research enterprise are automatically considered useless upon publication. A movement back to data sharing—with the understanding that not all flawed studies represent breaches of ethics—would also facilitate follow-up modeling and algorithm development and evaluation efforts designed to help the field progress, and to facilitate the overt comparative evaluation of alternative and new analytical approaches, which should make everyone’s studies better over time.

If authors fail to respond to reasonable requests for the data that is used to support a conclusion in a published study, or if they place unreasonable institutionalized strictures and policies in place for data sharing (e.g., protracted data sharing proposal submission/review processes), then informatics researchers should draft and send letters to the Editor of the journal in which they have published their conclusions requesting the data from the journal. Editors as well as authors have a responsibility for any negative attendant health consequences that works published in their journal might inflict on the public, and ease of data sharing should be rule, not the exception. Editors have options for enforcement; they can always offer to withdraw a paper that the scientific community cannot fully evaluate if the authors fail to comply with data sharing requests. Editors and publishers, however, have little incentive to police the integrity of the studies they publish, and perhaps the reputation of their journals are safest if they engage in such discourse only as a matter of last resort. Published letters to the editors inquiring on the status of the data requests are in keeping with good practices within an open scientific research community. Letters to funding agencies are a possible last resort.

Results reporting standards are needed more than data standards. I cannot enumerate all possible or desirable standards in a brief editorial, and perhaps should not due so because my writing is not peer reviewed and may not represent the consensus of the biomarkers and research informatics community. I will restrict my consideration to one type of results reporting. It is all too common in studies that use survivorship as an outcome to demonstrate that the predicted groups exhibit statistically significant survivorship curves. Any study that reports such a result should also be required to calculate and publish the ROC curves of each positive prediction made by a model that would lead to a change in the clinical treatment for any patient in the future. For example, a study that concludes that a treatment outcome prediction model works well because the difference in the predicted groups’ (responsive, not responsive) survivorship curves is statistically significant should also report estimates of the performance evaluation measures of each prediction type (e.g., SN, SP for ‘responsive’, SN, SP ‘not responsive’). They should then calculate the expected number of patients per year in a clinical group that, if the assay and model were in fact used on all patients qualified for the model, would suffer due to errors in the model. This step would go a long way to indemnify the medical practioners, kit manufacturers & insurance companies from litigation because the patient could then be informed of the full attendant risks in terms that they can understand. For example: “Of all of the people who could be given this test this year (4500), 1500 are expected to be told that they will respond to therapy. Of these 1500 patients, 35 are expected to not respond to therapy in spite of their prediction. Similarly, 3500 patients expected to be told that they might not respond to therapy. Of these 3500, 600 may in fact respond to therapy in spite of their prediction. You may or may not fall into either category of erroneously diagnosed patients and the risks for you, as an individual patient, are estimated as...”. Error rates on percentages will be useful for some patients, but some patients may not be able to envision that risk in terms of number of people per year. The full impact on the health of our population would also be made obvious with such simple reporting standards. This should empower the FDA to make more rapid conclusions about approval of a drug or an assay for clinical use, and for health care administrators to make informed decisions based on cost/benefit analysis grounded on real data.

Given the brevity of the treatment of these topics, I invite short papers on Publication Reporting Standards to Cancer Informatics. 

